# Consortium Framework Using Blockchain for Asthma Healthcare in Pandemics

**DOI:** 10.3390/s22218582

**Published:** 2022-11-07

**Authors:** Muhammad Shoaib Farooq, Maryam Suhail, Junaid Nasir Qureshi, Furqan Rustam, Isabel de la Torre Díez, Juan Luis Vidal Mazón, Carmen Lili Rodríguez, Imran Ashraf

**Affiliations:** 1 Department of Computer Science, University of Management and Technology, Lahore 54000, Pakistan; 2 Department of Computer Science, Bahria University, Lahore 54600, Pakistan; 3 School of Computer Science, University College Dublin, D04 V1W8 Dublin, Ireland; 4 Department of Signal Theory and Communications and Telematic Engineering, University of Valladolid, Paseo de Belén 15, 47011 Valladolid, Spain; 5 Higher Polytechnic School, Universidad Europea del Atlántico, Isabel Torres 21, 39011 Santander, Spain; 6 Department of Project Management, Universidad Internacional Iberoamericana, Arecibo, PR 00613, USA; 7 Project Department, Universidade Internacional do Cuanza, Cuito P.O. Box 841, Bié, Angola; 8 Department of Project Management, Universidad Internacional Iberoamericana, Campeche 24560, Mexico; 9 Department of Information and Communication Engineering, Yeungnam University, Gyeongsan 38541, Korea

**Keywords:** asthma, healthcare, blockchain, consortium framework

## Abstract

Asthma is a deadly disease that affects the lungs and air supply of the human body. Coronavirus and its variants also affect the airways of the lungs. Asthma patients approach hospitals mostly in a critical condition and require emergency treatment, which creates a burden on health institutions during pandemics. The similar symptoms of asthma and coronavirus create confusion for health workers during patient handling and treatment of disease. The unavailability of patient history to physicians causes complications in proper diagnostics and treatments. Many asthma patient deaths have been reported especially during pandemics, which necessitates an efficient framework for asthma patients. In this article, we have proposed a blockchain consortium healthcare framework for asthma patients. The proposed framework helps in managing asthma healthcare units, coronavirus patient records and vaccination centers, insurance companies, and government agencies, which are connected through the secure blockchain network. The proposed framework increases data security and scalability as it stores encrypted patient data on the Interplanetary File System (IPFS) and keeps data hash values on the blockchain. The patient data are traceable and accessible to physicians and stakeholders, which helps in accurate diagnostics, timely treatment, and the management of patients. The smart contract ensures the execution of all business rules. The patient profile generation mechanism is also discussed. The experiment results revealed that the proposed framework has better transaction throughput, query delay, and security than existing solutions.

## 1. Introduction

The COVID-19 pandemic has affected different aspects of life, in particular healthcare systems. Asthma patients are more vulnerable to being infected by coronavirus [1]. Asthma adds inflammation and narrowing of the lungs which restricts the air supply in muscle airway walls and produces mucus. Air walls are tubes with muscles [2], A person with asthma disease may experience wheezing, chest tightness, coughing, racing heartbeat, and breathlessness [3]. The asthma attacks begin suddenly and may result in the loss of life. In the case of swelling in the airways,  oxygen is prevented from reaching the lungs.

**Figure 1:**
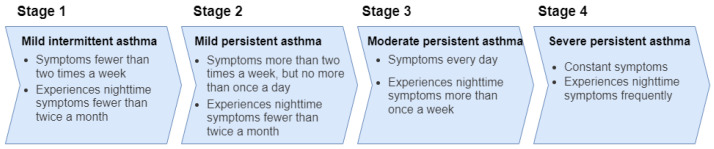
Stages of asthma.

Asthma patients approach hospitals mostly in critical conditions and require emergency treatment, which has been difficult during the COVID-19 pandemic [4]. Asthma disease can develop due to many different reasons, and the most common types of asthma are allergic asthma, non-allergic asthma, cough-variant asthma, and occupational asthma [5]. The four stages of asthma, mild intermittent asthma, mild persistent asthma, moderate persistent asthma, and severe persistent asthma, are shown in Figure 1.

**Figure 2:**
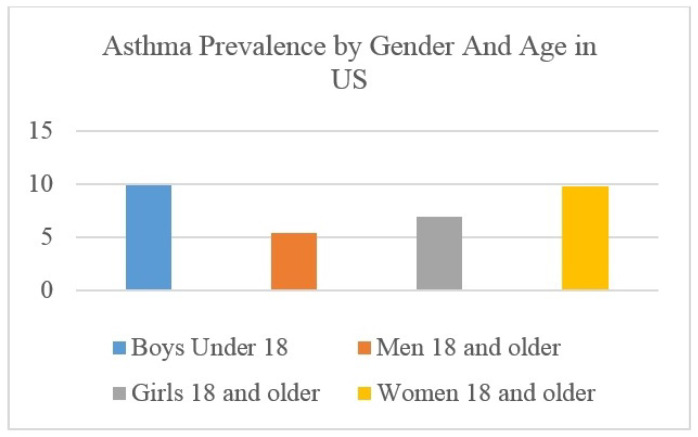
Asthma prevalence by gender and age in the USA.

According to the research on cross-sectional world health, approximately 21% of people in Australia, 20% in Sweden, 8.3% in the USA, 18.2% in the UK, and 15% in the Netherlands are affected by this disease [6]. After COVID-19, an increase in asthma patients has been recorded worldwide. Asthma disease is very common among males, females, and children. Asthma is most common during childhood [7]. The survey statics [8] in terms of gender and age are shown in Figure 2. This shows that boys and girls under the age of 18 and women aged18 and above are more vulnerable to the effects of asthma.

Coronavirus virus and its variants, such as Omicron, have symptoms that are very similar to asthma. Coronavirus and asthma both affect the lungs [9]. During the COVID-19 pandemic, many people suffered from asthma and lost their lives [10]. An increase in asthma patients was observed worldwide after COVID-19, which also created problems for data safekeeping. It is the responsibility of health institutions to keep the patients’ data safe from unauthorized/illegal access. Low- and middle-income countries are facing logistical challenges to identify the areas with a higher number of asthma patients to prioritize and plan treatment [11]. Therefore, it is important to make the records of patients secure and easily traceable by physicians for speedy diagnostics and timely treatment of patients. Most of the health systems in hospitals are based on a centralized database, and due to legal and technological constraints, patient data are not shared with other systems [12]. The trust and traceability of patient data comprise another requirement of stakeholders from the health system, which is not offered by current state-of-the-art systems.

The blockchain is a tamper-resistant digital ledger, which is implemented in a distributed manner without any centralized authority to avoid data altering after they have been published on the blockchain network [13]. As the blockchain is decentralized in nature, every node on the network keeps a record of the data, which provides more security and makes the system reliable. The data of the blockchain are transparent and traceable, which is basically a feature of the blockchain. This makes blockchain technology suitable for healthcare systems [14]. Smart contracts are automatically executable pieces of code that are stored on the blockchain. In a blockchain, all data and functions are carried out by events in smart contracts and consume much resource. Therefore, it is important to review the design [15] and conduct rigorous testing [16] of the smart contract code before deployment on the blockchain.

Blockchain technology securely shares healthcare records locally and globally helps physicians with speedy and better diagnostics in the event of an emergency during a pandemic. Developing countries are facing the challenges of identifying the areas that have the highest number of asthma patients to prioritize treatment. The patients in villages and remote areas do not have much knowledge about asthma disease and its basic treatment, which caused severe consequences during COVID-19. Mostly, patient history is not available to physicians due to many reasons such as patient consults with different doctors at many hospitals at different times and locations, which causes poor diagnostics and treatment. The handling and management of asthma patients can be improved using blockchain technology. Much work has been accomplished in healthcare departments related to asthma. However, there is no truly decentralized solution for asthma healthcare, specifically one that can operate during pandemic situations. In pandemics, it is critical for governments to keep track of hospitals and resources to provide the maximal services to the patients. There is a need for healthcare units, insurance companies, and the government to work together in pandemic situations while maintaining the privacy of patients’ data and early diagnosis of the disease state, which requires decentralized systems.

In this article, we propose an efficient, transparent, and secure blockchain consortium framework for asthma healthcare. The framework works on state-of-the-art blockchain technology, making it trustable, fast, and secure. The proposed framework overcomes emergencies for asthma patients in the case of a pandemic, such as COVID-19. The framework also facilitates asthma patients to receive medical and insurance services quickly. Moreover, the framework provides transparency in medical healthcare and makes traceability easier than already existing solutions. It allows governments to keep track of medical emergencies. The framework addresses the scalability issue of the blockchain with the decentralized Interplanetary File System (IPFS) technology to store patient data in encrypted form. The proposed framework was tested for data security, transaction throughput, query delay, and analysis of privacy protection. The data security experiment included secure patient profile creation with the help of a 12-word seed phrase and private and public keys. The transaction throughput and query delay experiment showed that the proposed framework is better at processing more transactions per day with an additional feature of less query delay. The novelty of our work is that it provides a complete solution to handle asthma patients in case of a pandemic including their medical needs, insurance policies, and complete data privacy.

The rest of the article is organized as follows. In Section 2, the related work is discussed. In Section 3, the proposed framework is discussed. Section 4 provides the experimental results and discussions. Section 5 consists of the conclusions and future directions.

## 2. Related Work

Anan et al. [17] proposed a health monitoring system for asthma patients by using Internet of things (IoT) devices. The proposed system remotely collects the healthcare data of the patient and transfers them to doctors for consultation. The system records the oxygen level, temperature, and heart rate of the patient in a remote area. The doctor can check the condition of the patient with the help of the mobile application. However, the data are collected by using the Android application, website, and sensor, which have security issues. Moreover, the system is centralized, and there was no defined policy for data sharing and traceability.

Woo et al. [18] proposed a healthcare application based on IoT systems to allow remote patient monitoring. The authors claimed the proposed application to be a reliable machine-to-machine IoT system for patients’ healthcare. However, the system is not transparent, and the usage of IoT devices increases the security threats. Moreover, the authors performed experiments using limited scenarios.

Zolnoori, M., et al. [19] investigated the process of asthma diagnosis and the treatment of asthma using artificial intelligence techniques. The methodology for constructing a fuzzy expert system for evaluating the amount of asthma exacerbation was also discussed in this research, which emphasized the necessity of the precise evaluation of inaccurate prescriptions and prompt treatment. However, the experiments performed were not good enough to prove data accuracy, and the system works on centralized systems. Another similar effort for patient data management was presented in [20] for hospitals’ data management, but the proposed system is centralized and relies on single-entity management. A single entity with all privileges can manipulate data in many ways and systems have no transparent access to data.

Tripathi et al. [21] proposed the blockchain-based smart healthcare system (SHS) framework, which provides security and transparency. However, the proposed framework is theoretical, and the authors did not perform any experiments to prove the system’s accuracy and security. Moreover, the authors did not discuss security attacks and other data integrity aspects. Chakraborty et al. [22] proposed a secure healthcare system design using blockchain technology. The data were collected through IoT devices and stored on the blockchain. However, the framework works on a hybrid storage and stores blockchain data on a centralized external storage, reducing security. This also causes a single point of failure. Moreover, privileged entities can control or manipulate the data. The business rules were also not implemented using smart contracts.

Chen et al. [23] discussed a framework for disease detection that is based on deep learning technology. It collects patient data by using wearable sensors and then analyzes the digital data for diagnosis. The framework is good for analyzing data. However, the framework is based on centralized servers, which reduces the data integrity and allows administrators to steal and manipulate data. Similarly, data traceability is also not possible.

In [24,25], the authors proposed multiple workflows that involved the ecosystem of healthcare by using blockchain technology for better management of patient data. The smart contract was used to develop a system that helps provide better health services and optimizes the costs. However, the proposed framework does not cover other stakeholders of the healthcare system. Similarly, it does not consider attack factors, and also, no experiments were performed to prove system security and quality.

These discussions reveal that much work has been carried out on healthcare departments and asthma. However, there is no complete decentralized solution for asthma patients, especially during pandemics. There is a need for a complete solution to increase the government’s abilities to keep track of the pandemic situation and available resources in multiple healthcare units. In this article, we propose a consortium framework for asthma patients. The novelty of this work is that it provides a complete solution to handle asthma patients in case of a pandemic including their medical needs, insurance policies, and complete data privacy. We also implemented encrypted IPFS data storage to increase data security and privacy to implement a scalable blockchain.

## 3. Proposed Framework for Asthma Healthcare System

**Figure 3:**
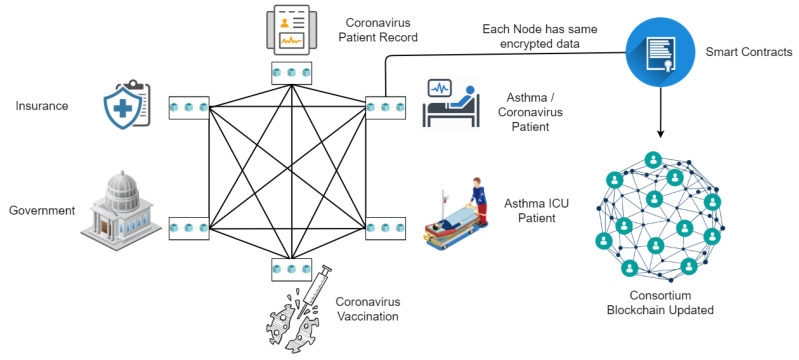
Blockchain consortium framework for asthma patient healthcare.

In this section, we propose an efficient, transparent, and secure blockchain consortium framework for asthma healthcare. This framework uses state-of-the-art blockchain technology and adds transparency to the system by allowing authorities to keep track of information. In addition, it ensures that patients’ data are encrypted and securely stored on the blockchain. Figure 3 shows how asthma patients’ health data can be managed efficiently using blockchain distributed ledger technology. The proposed framework connects compensation companies, coronavirus vaccination facilities, governments, healthcare data, and asthma patients. To transparently track asthma patient data, the government and other related sectors can be connected through this blockchain architecture. In the case of a pandemic situation, the prioritization of asthma patients becomes easier, and they can be admitted quickly. Due to the connectivity of all stakeholders, it will be easier for asthma patients to make compensation claims to insurance companies, get help from the government, and track their health status more transparently.

### 3.1. Layer Structure of Proposed Framework

**Figure 4:**
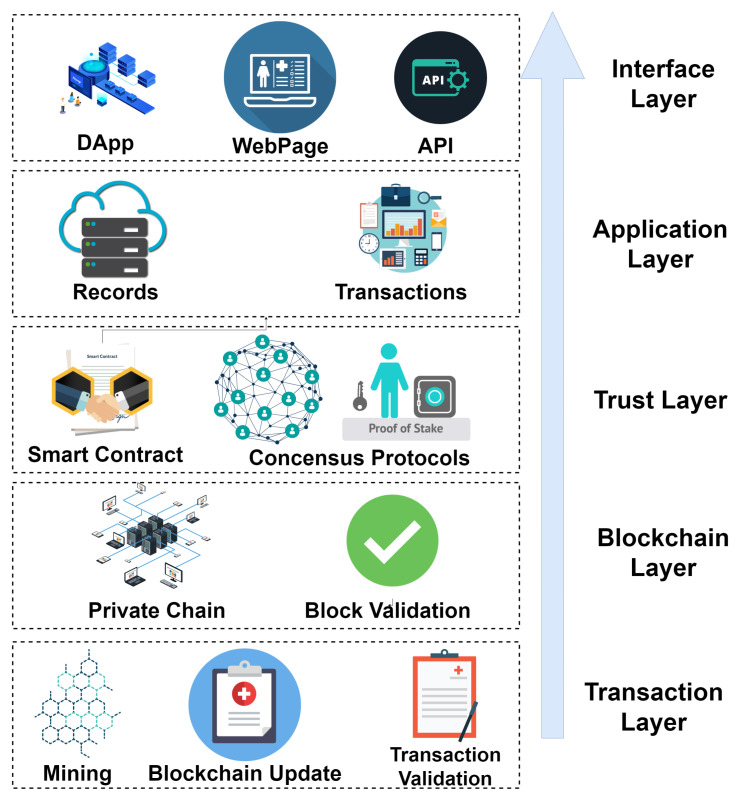
Layered architecture of blockchain consortium.

In this section, we define five layers of the blockchain consortium framework. Figure 4 shows the five layers of the consortium blockchain framework, and a brief overview is provided here:

1. Layer 1: This is the interface layer and is considered the first layer, which directly interacts with users. This layer includes blockchain-based decentralized applications (DApps), a web interface, and application programming interfaces (APIs), which patients and healthcare staff use for interacting with the system.
2. Layer 2: The application layer includes transactions and records that are generated by the interaction of users with the provided interfaces. This layer is responsible for arranging data and records for input into the blockchain.
3. Layer 3: This is the trust layer, which gains trust between consortium nodes for asthma healthcare. This layer is responsible for providing a trusted connection between compensation companies, coronavirus vaccination facilities, governments, asthma patients, and healthcare data. It includes smart contracts and the consensus protocols of the blockchain. The proof of stake (PoS) was used as a consensus protocol due to its reliable and fast response.
4. Layer 4: This is the blockchain layer, which deals with the consortium and private blockchains for the transactions. Block validation adds new transactions to the blockchain after validating the block from all peer nodes. By default, the framework uses a consortium blockchain, which allows anyone to see the transactions, making it completely transparent.
5. Layer 5: This is the transaction layer, which responsible for mining and transaction validation. This layer ensures that each new block added to the blockchain is valid. This layer updates the state of the blockchain.

### 3.2. Proposed Framework Architecture and System

**Figure 5:**
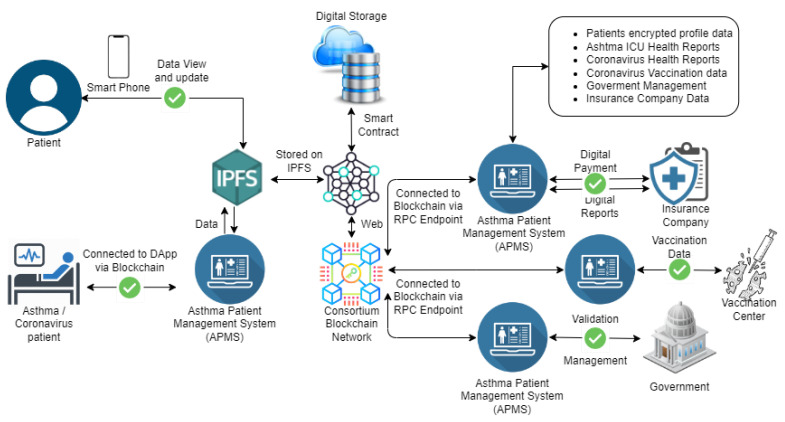
Peer-to-peer connectivity blockchain consortium framework for asthma patient healthcare.

The architecture of the proposed consortium framework is presented in Figure 5. The main stakeholders of the proposed framework are asthma and coronavirus patients, insurance companies, vaccination centers, ICU laboratories, and the government. A new blockchain-based system is introduced under the name of the asthma patient management system (APMS). This system deals with the following data:

1. Patients’ encrypted profile data;
2. Asthma ICU health reports;
3. Coronavirus health reports;
4. Coronavirus vaccination data;
5. Government management data;
6. Insurance companies data.

The APMS allows patients to access their personal information through mobile apps or web apps. The patient can view and update data and contact authorities for assistance and help. The APMS saves patients’ data in encrypted form, making it secure and hack-proof from all possible attacks and only allows the patient to decrypt them by utilizing their private keys. Figure 5 shows that the encrypted data are stored on the Interplanetary File System (IPFS) with the help of smart contracts, and the blockchain keeps a record of the hash values of the uploaded data. The hash value of each of the data is designed in such a way that, if someone tries to alter even 1 bit of the data, then it changes the hash completely, which makes it easy to track if someone has tried to alter the data. The APMS is also connected to ICU laboratories, which have their registered profiles on the APMS. These laboratories can add patient data and update them according to their health reports. These reports are also stored in encrypted form, and their backups are uploaded on the IPFS to ensure their decentralized availability. The blockchain makes it easy for users to track information, but it does not allow them to read personal healthcare information.

The vaccination centers keep track of patient profiles on the APMS and update each patient’s vaccine information. The insurance companies also interact with the APMS to obtain any patient’s information and proceed with insurance claims. Government entities have the authority to check patients’ data to keep track of asthma and coronavirus to take countermeasures for controlling them. Emergency healthcare services receive notifications from the APMS in case of an emergency, and patients also have the option to inform about an emergency from their mobile application.

### 3.3. Data Encryption and Security

**Figure 6:**
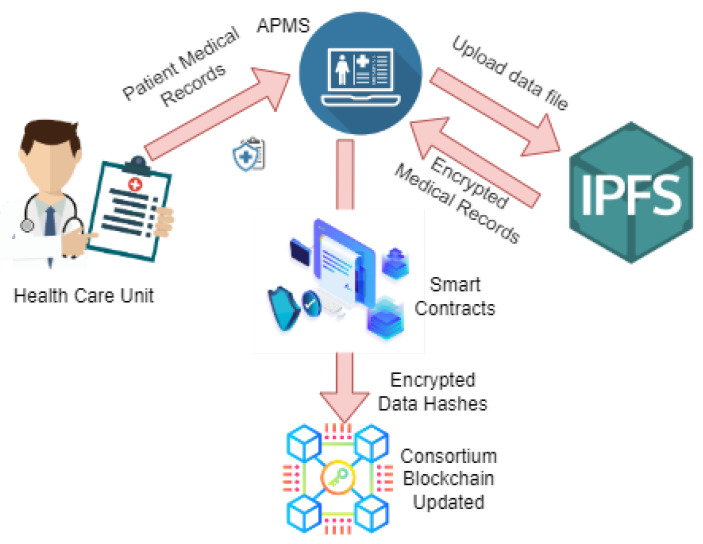
Workflow of patients’ medical records through the APMS.

The patient medical record is considered to be personal information and cannot be shared publicly. Hence, to maintain data integrity and security, the APMS utilizes IPFS services and encryption. First of all, the data are uploaded to the APMS by the medical healthcare units. The APMS divides the big data files into small chunks and encrypts them with individual private keys. Then, these encrypted data chunks are uploaded to the IPFS’s decentralized storage, and the hash values of each of the data are achieved. These data hash keys are stored on the blockchain with the help of a smart contract. This process maintains data integrity and decentralization. Figure 6 depicts the workflow of patient medical records through the APMS with the help of smart contracts and the IPFS.

### 3.4. Smart Contracts

**Figure 7:**
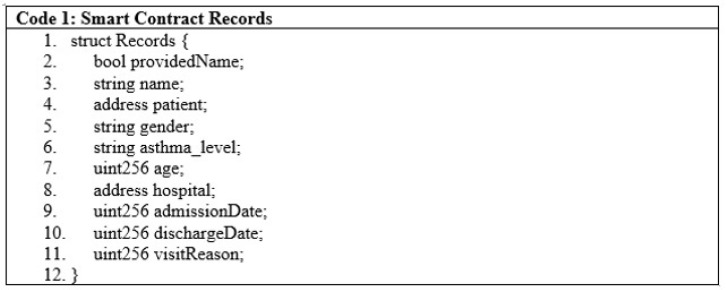
Smart contract records.

Smart contracts are a finite piece of code that is included in transactions and stored on the blockchain. Smart contract users interact with them by calling multiple functions through transactions. These functions can either change the state of the blockchain or just read the data. The asthma healthcare system is made up of a smart contract that is EVM-compatible and is written in the solidity programming language. This smart contract is capable of keeping a record of patient stay conditions, symptoms, hospital admission dates, and discharge dates, as shown in Figure 7, lines 1 to 11.

**Figure 8:**
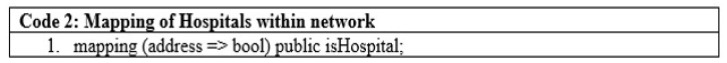
Mapping of hospitals within the network.

All hospitals within the network are mapped according to their public addresses, and it can be seen if a hospital is included in the network or not, as illustrated in Figure 8, code line 1.

**Figure 9:**
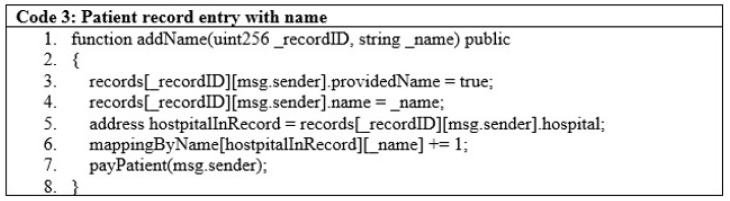
Patient record entry with name.

Patients’ data are entered based on their name and identity and passed through the add name function as given in line 1 of Figure 9. The minimal code for patient data is shown in Figure 9, lines 3 to 7.

**Figure 10:**
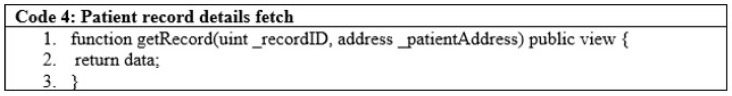
Patient record detail fetching.

Once a record is stored in the APMS, the stakeholders can access the data by searching either the names or id numbers of the patient, as shown in Figure 10.

**Figure 11:**
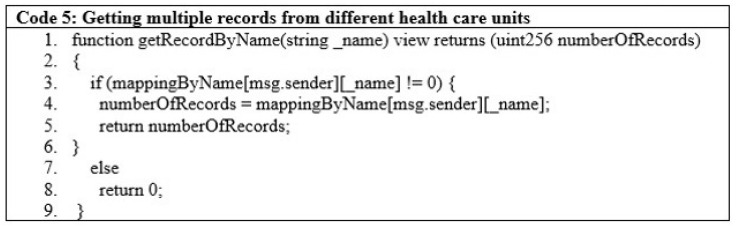
Obtaining multiple records from different healthcare units.

Healthcare departments can fetch multiple records for patients by searching through their names, as in Figure 11. These records can be from other healthcare units as well. The condition on line 3 checks if the sender’s information is available in the system.

**Figure 12:**
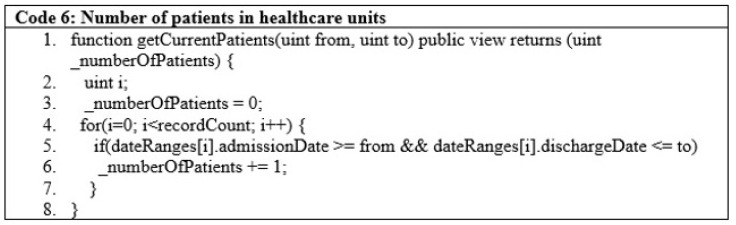
Number of patients in healthcare units.

The government can also check the total number of patients staying in healthcare units and their details, as depicted in Figure 12, code lines 1 to 8.

### 3.5. Execution of Smart Contract

**Figure 13:**
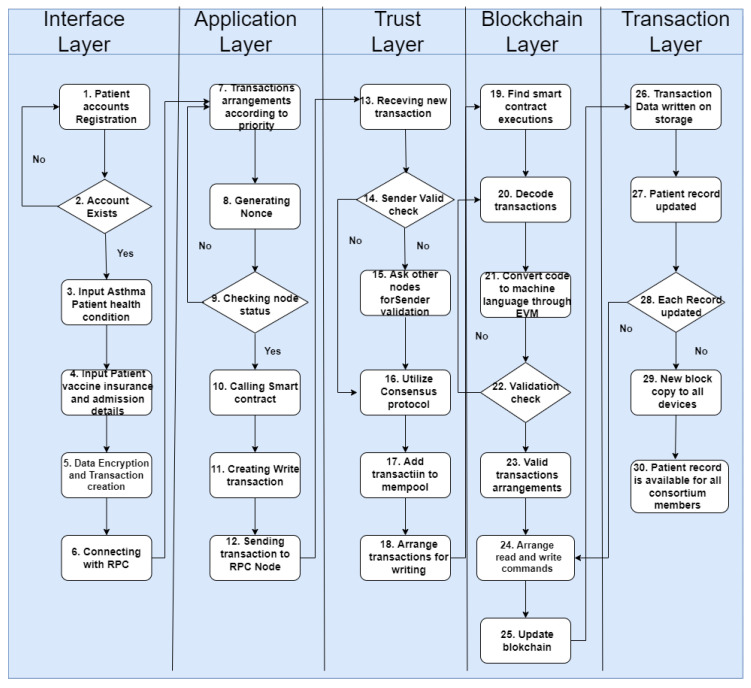
Flowchart of smart contract execution.

The flow of the proposed framework is illustrated in Figure 13. The user enters the input, which includes the total number of asthma patients, patients assigned a blockchain address, the government healthcare department assigned address, the blockchain address assigned to hospitals, and the address assigned to the insurance companies. Once the input has been performed, the process of the system will start. In the first step, the patient registers a new account, and if there is already an account, then the request is not processed; otherwise, an account is created. Then, asthma patients’ health conditions are entered into the system. After that, the vaccination, insurance, and admission details of the patient are recorded. In the fifth step, the data are encrypted and a transaction created. In the sixth step, a connection with RPC is built. In the seventh step, transactions are arranged according to priority. In the next step, the nonce number is generated according to transaction numbers. In the ninth step, the node status is checked, and if it is not validated, then the process moves back to Step 7. Next, a smart contract is called and a written transaction generated. After that, the transaction is sent to the RPC node, followed by the receiving of the transaction at the node end. The transaction sender is verified of whether it is valid or not. After that, the sender’s validity is checked. In Steps 16 and 17, the consensus protocol is utilized and the transaction added to mempool. In the next step, transactions are arranged and sent for writing. In Steps 19 and 20, smart contract executions are founded and transactions decoded. In the next step, the code is converted to machine language through EVM. Step 22 checks for validation, which is followed by arranging valid transactions. In steps 24 and 25, commands are arranged for reading and writing, and the blockchain is updated. In the next step, the transaction data are written and saved on the blockchain permanently. Patient records are updated in Step 27, while Step 28 checks if each record is updated correctly. The last two steps send data copies to all devices and make sure that they are available to all consortium members.

### 3.6. The 51% Attack

**Figure 14:**
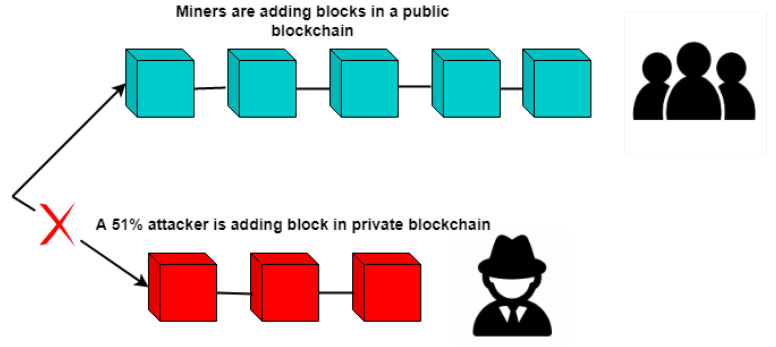
The 51% attack.

The 51% attacks happen when a miner gains a 51% hash rate of the blockchain, and then, it can use this hash power to manipulate the blockchain by adding malicious blocks. In the proposed framework, two solutions are provided to overcome the 51% attack vector. First, the consensus algorithm makes sure that no miner possesses a 51% hash rate. The second solution to prevent attacks is to maintain a repository for trustable miners by government authorities. These validated miners will need to provide the verification details to authorities in order to mine blocks for the consortium framework. This also ensures that no external miner is allowed to add blocks to the blockchain. If a verified miner tries to manipulate the blockchain information somehow, then penalties are implied. Figure 14 illustrates an example of a 51% attack where an attacker connects to the blockchain, copies data, and then disconnects. The attacker then mines the private chain and then connects back to the network with a longer chain.

### 3.7. Chain Security

The proposed framework provides chain security through consensus algorithms, as shown in Algorithm 1. These algorithms work on all nodes that mine blocks on the blockchain. If a node adds a block, then the algorithms compare the new node hash with the previous node hash, and if it is a valid block, it is added to the blockchain and sent to all other peer nodes. If a node adds an invalid block, then the algorithms check its hash with the previous block on the blockchain and rejects it. Then, this invalid node is disconnected from the network, while the remaining nodes keep adding new blocks to the blockchain. When this node is added back to the network, the longest chain is replicated automatically by the algorithms.
**Algorithm 1** Chain security algorithm.**Input:** current_block**Output:** Bool True  1:**Func**(Hash)  2:**Input** Block: Timestamp, previous_hash, nonce, data  3:     SHA256(block)  4:     **return** Hash  5:**End Func**  6:**Initialize** previous_block = 0 index  7:**Initialize** block_index = 1  8:**while** block_index is less than length of the chain **do**  9:   Get current_block = block_index10:   **if** previous_hash! = hash of block_index −1 **then**11:       **return** False12:   **end if**13:   previous_proof = proof of previous_block14:   proof = proof of current_block15:   has_operation = sha256(proof-previous_proof)16:   **if** hash_operation’s first four digits 000 **then**17:       **return** false18:   **end if**19:   previous_block = current_block20:   Block_Index = Block_Index + 121:   **return** True22:**end while**

**Figure 15:**
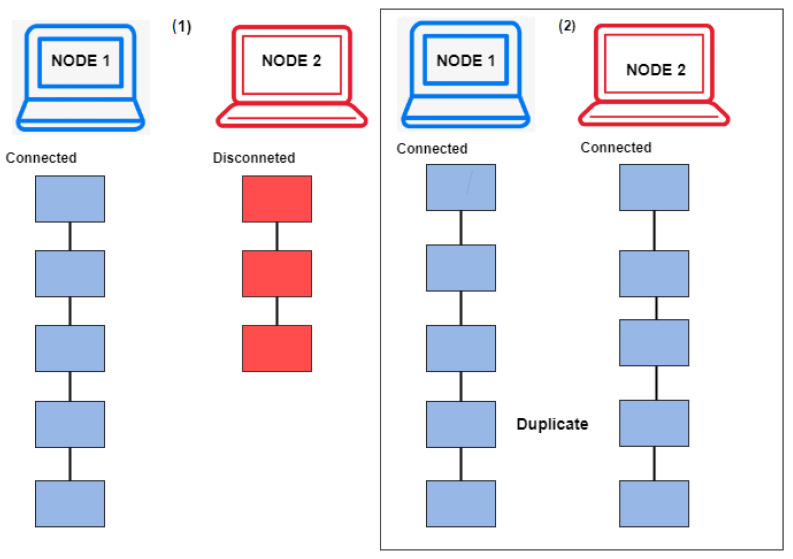
On-chain block replication on the blockchain network.

Figure 15 illustrates how Node 2 gets disconnected from the network while Node 1 keeps mining blocks, and later, when Node 2 joins the network back, the block data are replicated to Node 2 as well, due to the longest chain algorithm rule.

### 3.8. Transactions in Proposed Framework

**Figure 16:**
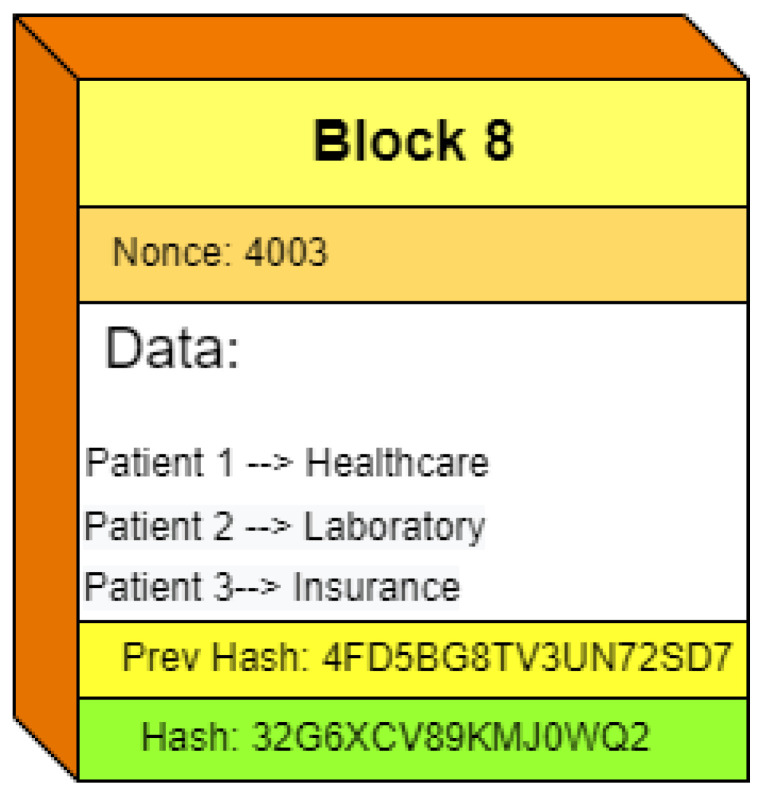
Block example with UTXO.

The unspent transaction output (UTXO) for our proposed framework provides information changes after transactions’ addition on the blockchain. In the proposed framework, UTXO contains transaction data such as patient information, insurance changes, medical status, and other details. Figure 16 illustrates an example block structure. It has a block number that indicates the number of new blocks in the whole blockchain. The nonce number is required for block mining. The data section includes transactions and the UTXO of the proposed framework. The block also shows the hash of the previous block and the hash of the current block.

### 3.9. Flexible Consensus Algorithm

The proposed framework consists of a flexible consensus, which helps to make the blockchain scalable and more secure. The proposed framework has the capability to change the consensus protocols at the time of blockchain deployment. The algorithm selected for testing purposes was the proof of stake (POS), in which validator nodes can confirm transactions and add them to the blockchain, while if a node adds false transactions, then it loses its staked coins. The flexible consensus provides security and scalability. The consensus algorithm can be changed according to the needs and requirements to increase performance. Other consensus algorithms that can be used for the consortium framework blockchain are the proof of work, proof of vote, ripple, and proof of trust.

## 4. Experiments and Results

The proposed framework was based on the POS. For the experiments, the Ubuntu 20.04 operating system, Canonical Group Limited, Isle of Man, United Kingdom, was used. The blockchain was programmed using C, C++, and Python, while the higher layer was supported by HTML5 and JavaScript. The smart contracts were written in the Solidity language. For blockchain node deployment, a virtual private server was used with the hardware configuration of an 8-core CPU, 1 TB SSD, and 16 GB RAM. The Internet upload/download speed was required to be maintained at a minimum of 1 MB/s. For testing purposes, only one node was used for validating the transactions. The blockchain network was started from a genesis block. The average block size was around 0.7 Kb. The blockchain size was increased from 0 Kb to 242.12 Kb at 500 blocks, while the average block time was 3 s.

### 4.1. Patients and Other Stakeholder Profiles

The APMS generates patient and other stakeholder profiles using their data. Each stakeholder provides personal verification information for registration on the APMS. This information is gathered and then verified by cross-matching from national identity resources, and once information is confirmed, a mnemonic seed phrase is generated. This seed phrase consists of 12 words, and it is very personal information, as anyone with these 12 words can access all information stored on the APMS for a user. The seed phrase consists of specific bep-0039 words [26] and cannot be made apart from these words. This seed is used for the generation of root keys through the m/44’/60’/0’/0/0 path, which leads to the generation of private and public keys to the wallet. Table 1 illustrates an example account with its seed, root key, private key, public key, and address.

**Figure 17:**
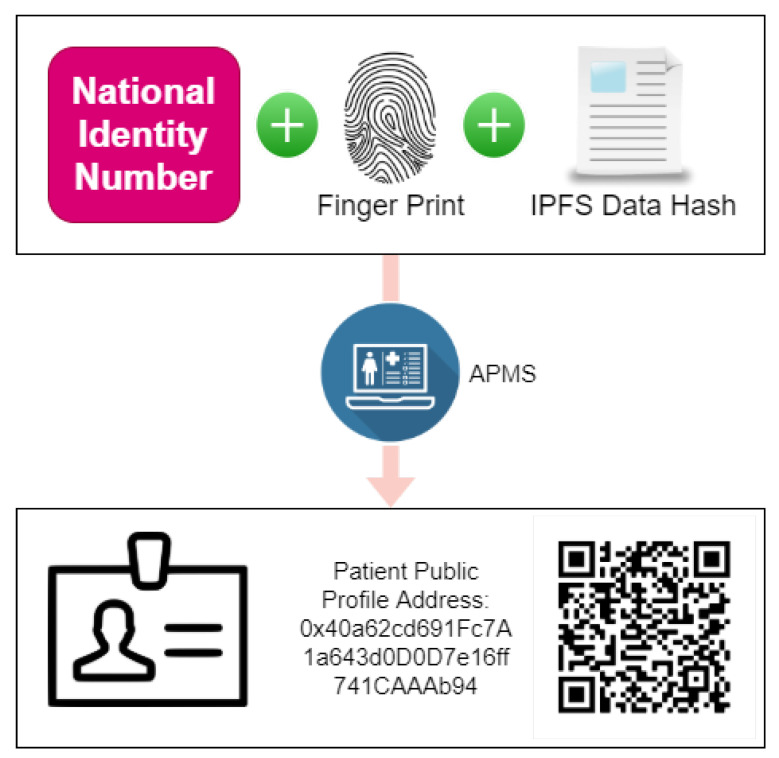
Patient profile generation process.

Each patient account is linked with fingerprints, national IDs, and the hash key of the personal information, and a QR-based profile is generated. The QR code can be scanned to gain easy access to basic public information of the user and then search for medical records. Figure 17 illustrates the patient profile generation process.

### 4.2. Transaction Throughput

The proposed framework deals with pandemic and asthma disease patients, so it is very critical for the proposed framework to be scalable and have more transaction-serving capabilities. The Apache Jmeter software was used for analyzing the transactions for a duration of 7 days over a test network. The collected data transactions per hour (TPHs) of the proposed framework were calculated with the average formula, and the transactions per second (TPSs) were calculated by converting hours into seconds.

(1) $$TPH=\left(\frac{\sum_{x=7}^{x=1}\mathrm{Transactions}\;\mathrm{of}\;\mathrm{Day}(x)}{\mathrm{Total}\;\mathrm{number}\;\mathrm{of}\;\mathrm{days}}\right)/24$$

(2) $$TPS=\frac{TPH}{3600}$$

**Figure 18:**
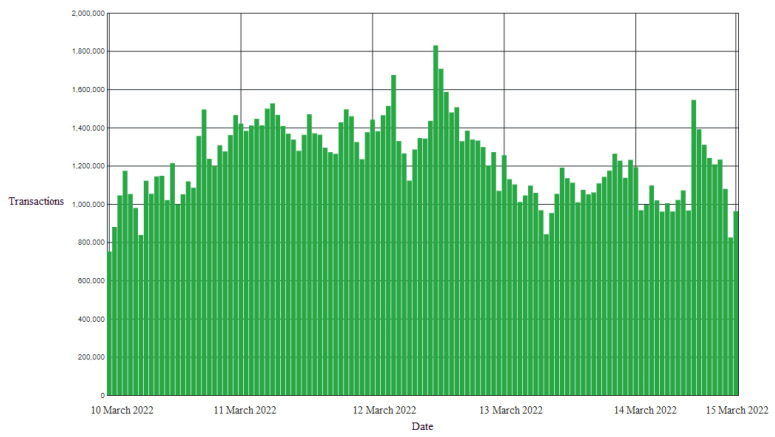
TPHs of the proposed framework.

Figure 18 illustrates the transactions per hour data of the proposed framework. By utilizing the TPH equation, the results for the proposed solution indicated 416,666.6 TPHs, and by utilizing the TPS equation, a result of 60 TPSs was achieved. The number of average transactions of the proposed framework was 60 transactions per second. The TPHs of the framework were between 800k and 1.8 million. This shows that the proposed framework can handle a large number of requests/transactions in a day. In the case of a pandemic, the proposed framework can handle the maximum requests on an hourly basis, making it best in performance.

Table 2 shows the TPS comparison of the proposed framework with other blockchain solutions and also calculates the transactions per day and the energyfee for each transaction. Bitcoin has TPSs of 7, and Ethereum has 20 TPSs. Due to PoW consensus protocols, both of these solutions have low TPSs. However, the proposed solution was better with TPSs of 60 and can perform better due to its PoS nature. The energyfee per transaction was very low compared to other existing solutions. The government can reduce the energyfee to zero by launching its own blockchain network instead of using a publicly available solution such as Ethereum and the Binance smart chain.

### 4.3. Query Delay

**Figure 19:**
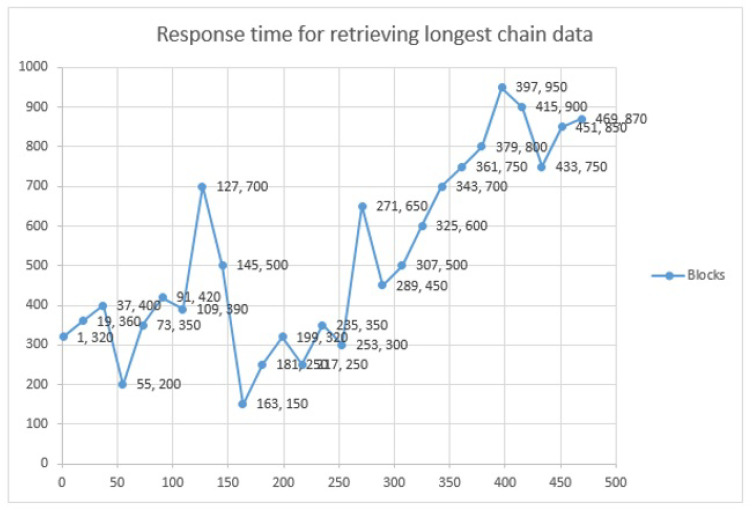
System response on user queries.

The delay of user queries for the proposed framework is depicted in Figure 19. The *x*-axis shows increasing nth queries in the blockchain, while the *y*-axis shows the query delay latency in milliseconds. In the proposed framework, users mostly query the data, and due to its blockchain nature, only the current state of the blockchain data is read, which returns the encrypted hash value of the IPFS data. The average latency was 350 milliseconds without any delay and long network congestions. The data were collected by running our own node.

### 4.4. Analysis of Privacy Protection

Any of the data uploaded to the blockchain are public, and anyone can read them. Patients’ data are very personal, and is critical if someone can gain access to them. The proposed framework stores the encrypted hash values of the IPFS uploaded data on the blockchain. Therefore, in case anyone gains access to the hash value of uploaded data on the blockchain, he/she still cannot gain access to the original file to the hash SHA-256 encryption, making the proposed framework best for privacy protection.

### 4.5. Analysis of Results

The experiments performed for the proposed framework showed that the proposed framework improved the performance over previously available solutions. The proposed framework had a better privacy mechanism to store patient information securely and safely. The proposed framework can execute an average of 60 transactions per second, which is faster than previously available solutions. The latency of the proposed framework and the average cost of the transaction were also lower than other competitive solutions. However, there are some limitations, as well, which we intend to solve in the future. Blockchain technology is ever-increasing in complexity, and it is important to keep pace with the latest security protocols. Especially in a medical context, it is important to keep up with the latest security features. The proposed framework can be developed in a better way with a continuous approach, as discussed in [28].

### 4.6. Discussions and Limitations

The results showed that the proposed framework was better than the previous solutions in terms of speed, cost, and safety. However, the cost can further be reduced if consortium stakeholders decide to run their own blockchain instead of using a publicly available blockchain. The transaction fee can be reduced to zero, and trusted entities can run mining nodes for the blockchain. However, this cost reduction method is vulnerable and can introduce compromises in the security of the system, as it leads to promoting the centralization path.

## 5. Conclusions

This paper proposed a consortium framework for asthma healthcare units. Coronavirus and asthma patients have similar symptoms, which causes problems for healthcare units in handling asthma patients. The proposed framework connects asthma healthcare units, coronavirus patient record centers, insurance companies, coronavirus vaccination centers, and the government through the secure blockchain network. The proposed framework helps asthma patients gain easy access to healthcare ICU units and other departments, in case of pandemics, as their data are arranged and available for the staff. It also helps in the timely provision of vaccines and insurance claims, as they are also connected to the same framework. The government can also keep track of the pandemic situation and available resources. The layered architecture and smart contract code and execution were discussed in detail. The patient profile creation and data encryption techniques were also discussed for data safety and integrity. The experimental results showed that the proposed framework performs better than existing solutions in terms of data security, transaction throughput, query delay, and privacy. In the future, we have plans to include other diseases in our consortium framework and make it a complete pandemic control and tracking system. Future work of this research is to work on dispersing the storage of patient data through chunks on the IPFS to enhance security. Smart contracts can also be optimized to reduce energyconsumption.

## Figures and Tables

**Table 1:** Patient credential attributes.

Key	Value
Seed	Palm depend road canvas scorpion wide great spike pitch trust shallow lesson
Root Key	xprv9s21ZrQH143K34gcEEcV78HDwBAu4PohzbnfxswwQBrcpzNY6c2phmy WMMM1pQEwjEbfqSJxspq2DnLNouK4wFYauc8KvuYp41Gguc7bfvh
Private Key	0xd9e9aa6212b635b6787c78f2c7f57d3f1ca92c2a383a9e82af74e09ae9a3b3c3
Public Key	0x03bf260041536e4765cc2a1b20fa1e4b75d75bb9d86d40b702fe6c75c435cdd8bc
Address	0x40a62cd691Fc7A1a643d0D0D7e16ff741CAAAb94

**Table 2:** TPS comparison with other blockchain solutions.

Blockchain	Transactions per Second	Fee per Transaction (USD)	Transactions/Day
Satoshi et. al. [13]	7	USD 10	604k
Vitalik et. al. [27]	20	USD 35	1728k
Proposed Framework	60	USD 0.3	5184k

## Data Availability

Data is available from the authors upon request.
